# Cycling area can be a confounder and effect modifier of the association between helmet use and cyclists’ risk of death after a crash

**DOI:** 10.1038/s41598-022-07135-1

**Published:** 2022-02-24

**Authors:** Daniel Molina-Soberanes, Virginia Martínez-Ruiz, Daniel Águila Gordo, Luis Miguel Martín-delosReyes, Mario Rivera-Izquierdo, Pablo Lardelli-Claret

**Affiliations:** 1 Department of Preventive Medicine and Public Health, School of Medicine, University of Granada, Avenida de la Investigación 11, Building A, 8th Floor, CP 18016 Granada, Spain; 2 Centre for Biomedical Research in Network of Epidemiology and Public Health (CIBERESP), Madrid, Spain; 3 Health Research Institute of Granada (Instituto Biosanitario de Granada, ibs.GRANADA), Granada, Spain; 4 Cardiology Service, University General Hospital, Ciudad Real, Spain

**Keywords:** Risk factors, Public health, Epidemiology

## Abstract

The effect of helmet use on reducing the risk of death in cyclists appears to be distorted by some variables (potential confounders, effect modifiers, or both). Our aim was to provide evidence for or against the hypothesis that cycling area may act as a confounder and effect modifier of the association between helmet use and risk of death of cyclists involved in road crashes. Data were analysed for 24,605 cyclists involved in road crashes in Spain. A multiple imputation procedure was used to mitigate the effect of missing values. We used multilevel Poisson regression with province as the group level to estimate the crude association between helmet use and risk of death, and also three adjusted analyses: (1) for cycling area only, (2) for the remaining variables which may act as confounders, and (3) for all variables. Incidence–density ratios (IDR) and their 95% confidence intervals were calculated. Crude IDR was 1.10, but stratifying by cycling area disclosed a protective, differential effect of helmet use: IDR = 0.67 in urban areas, IDR = 0.34 on open roads. Adjusting for all variables except cycling area yielded similar results in both strata, albeit with a smaller difference between them. Adjusting for cycling area only yielded a strong association (IDR = 0.42), which was slightly lower in the adjusted analysis for all variables (IDR = 0.45). Cycling area can act as a confounder and also appears to act as an effect modifier (albeit to a lesser extent) of the risk of cyclists’ death after a crash.

## Introduction

The hypothesis that helmet use decreases the risk of death in cyclists involved in road crashes has been supported in most previous works, including recent meta-analyses^1,2^. However, the magnitude of this association varies widely across studies, some of which in fact reported an inverse association. For example, in a Spanish study that applied a Bayesian network model to a national police-based database, Aldred et al.^3^ found a higher risk of death or severe injury after a crash in helmeted cyclists compared to non-helmeted ones (relative risk = 1.3). Among the reasons for the discrepancies among studies (which may include, but are not limited to, small sample size, e.g., no large studies have estimated negative effects of helmet use on head injury), confounding and effect modification stand out. These are two theoretically different concepts: confounding is a bias which must be removed; effect modification reflects a real biological effect which should be detected and communicated^4^.

With regard to confounding, it seems clear that helmeted and non-helmeted cyclists differ in many other characteristics which may affect their risk of death after a crash, and these differences preclude an unbiased estimate of the possible causal association between helmet use and death^5–9^. Unfortunately, in a recent meta-analysis highlighting the protective effect of helmet use by cyclists, Høye^1^ found that adjusting for confounders was uncommon: researchers adjusted mostly for age or sex, if at all. However, cyclist- or environment-related characteristics may influence the strength of the true causal relationship between helmet use and death, acting as effect modifiers. An example of this phenomenon is the speed of the collision. For example, it could be hypothesized that the protective effect of helmets will be lower at slower speeds of collision, because in this situation the risk of death approaches 0 independently of helmet use.

**Figure 1:**
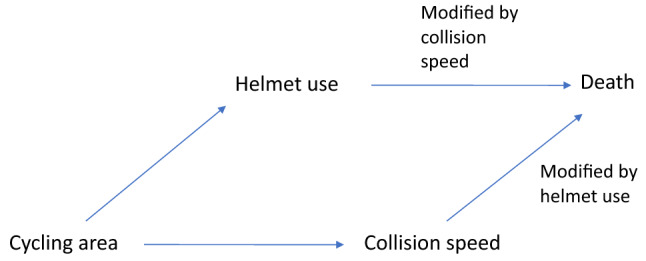
Directed acyclic graph of the theoretical model for the confounding or modifier effect of cycling area on the causal path between helmet use and death.

It is not easy to find published examples of actual variables which should be considered potential confounders or effect modifiers. Cycling area (open road vs. urban setting) is an excellent example of a variable which can act a priori as both as a confounder and (indirectly) as an effect modifier in the causal link between helmet use and death. The directed acyclic graph depicted in Fig. 1 illustrates this dual role. Helmet use is mandatory in Spain for cycling on open roads but not in urban areas (except for children under 16 years old). Therefore, the distribution of cycling area is clearly unbalanced between helmeted and non-helmeted cyclists involved in road crashes. Furthermore, cycling area strongly affects the travelling speed of both cyclists and other vehicles on the road, which in turn is the main determinant of cyclists’ risk of death after a collision. These facts make cycling area a potentially strong confounder, opening a backdoor (non-causal) path between helmet use and death, and thus biasing toward the null any estimates of the causal path. Furthermore, cycling area is a major ascendant variable that influences collision speed by setting speed limits for each of the two environments. Therefore, the speed at the time of the crash would be related to the amount of kinetic energy at impact^10^ and, as hypothesized above, may in turn modify the magnitude of the causal association between helmet use and death.

The aim of this study is to search for evidence for or against the hypothesis that cycling area may act as a confounder and an effect modifier of the association between helmet use and risk of death among cyclists involved in road crashes.

## Results

Descriptive information for all study variables is presented in Table 1. Table 2 shows all incidence–density ratios (IDR) estimated to assess the association between helmet use and risk of death among cyclists. Crude IDR estimation yielded a point estimate of 1.10, but this ratio was less than 1 when it was calculated separately for the two strata defined by cycling area. This inverse association was substantially stronger for cycling on open roads than in urban settings, with a *P* value of 0.024 for the interaction term. After adjustment for all possible confounders except cycling area, IDR showed a moderate inverse relationship between helmet use and risk of death (0.81), but the 95% CI clearly included the null value. Again, stratification of this value according to cycling area revealed a similar pattern to that found in the crude analysis, although with a smaller difference between the two estimates and a higher *P* value for the interaction term (0.223). When IDR was adjusted only for cycling area, it showed a strong inverse association (0.42; 0.31–0.58), which was only slightly weaker after adjustment for all variables (0.45; 0.32–0.63).

**Table 1:** Distribution of the study variables in the sample of cyclists, stratified by cycling area.

Variable	Categories	All		Urban areas		Open roads	
		N	%	N	%	N	%
	Total	24,605	100	16,574	100	8031	100
Death	No	24,263	98.61	16,408	99.00	7855	97.81
	Yes	239	0.97	65	0.39	174	2.17
	Unknown	103	0.42	101	0.61	2	0.02
Helmet use	No	6932	28.17	6068	36.61	864	10.76
	Yes	11,083	45.04	4603	27.77	6480	80.69
	Unknown	6590	26.78	5903	35.62	687	8.55
Sex	Male	20,321	82.59	12,959	78.19	7362	91.67
	Female	3926	15.96	3266	19.71	660	8.22
	Unknown	358	1.45	349	2.11	9	0.11
Age groups (years)	< 10	299	1.22	270	1.63	29	0.36
	≥ 10–15	1045	4.25	909	5.48	136	1.69
	≥ 15–20	2453	9.97	2070	12.49	383	4.77
	≥ 20–25	1895	7.70	1606	9.69	289	3.60
	≥ 25–30	1928	7.84	1510	9.11	418	5.20
	≥ 30–35	2183	8.87	1526	9.21	657	8.18
	≥ 35–40	2608	10.60	1601	9.66	1007	12.54
	≥ 40–45	2467	10.03	1433	8.65	1034	12.88
	≥ 45–50	2187	8.89	1208	7.29	979	12.19
	≥ 50–55	1872	7.61	1071	6.46	801	9.97
	≥ 55–60	1494	6.07	821	4.95	673	8.38
	≥ 60–65	1114	4.53	543	3.28	571	7.11
	≥ 65–70	913	3.71	444	2.68	469	5.84
	≥ 70–75	523	2.13	253	1.53	270	3.36
	≥ 75	511	2.08	304	1.83	207	2.58
	Unknown	1113	4.52	1005	6.06	108	1.34
Nationality	Spanish	17,936	72.90	11,757	70.94	6179	76.94
	Foreign	651	2.65	230	1.39	421	5.24
	Unknown	6018	24.46	4587	27.68	1431	17.82
Reason for cycling	Work-related	1014	4.12	818	4.94	196	2.44
	Other reason	10,899	44.30	5014	30.25	5885	73.28
	Unknown	12,692	51.58	10,742	64.81	1950	24.28
Cycling infractions	No	10,555	42.90	6211	37.47	4344	54.09
	Yes	4315	17.54	3116	18.80	1199	14.93
	Unknown	9735	39.57	7247	43.73	2488	30.98
Type of crash	Collision with moving vehicle	14,143	57.48	9930	59.91	4213	52.46
	Other	10,462	42.52	6644	40.09	3818	47.54
Road surface	Normal	21,306	86.59	14,102	85.09	7204	89.70
	Altered	1983	8.06	1178	7.11	805	10.02
	Unknown	1316	5.35	1294	7.81	22	0.27
Weather conditions	Good	20,061	81.53	12,707	76.67	7354	91.57
	Bad	1570	6.38	974	5.88	596	7.42
	Unknown	2974	12.09	2893	17.46	81	1.01
Traffic lane characteristics	Intersection	10,075	40.95	7225	43.59	2850	35.49
	Other	14,530	59.05	9349	56.41	5181	64.51
Time of day	00:00–02:59	343	1.39	299	1.80	44	0.55
	03:00–05:59	152	0.62	126	0.76	26	0.32
	06:00–08:59	1959	7.96	1384	8.35	575	7.16
	09:00–11:59	5938	24.13	3054	18.43	2884	35.91
	12:00–14:59	5765	23.43	3670	22.14	2095	26.09
	15:00–17:59	3812	15.49	2820	17.01	992	12.35
	18:00–20:59	4931	20.04	3715	22.41	1216	15.14
	21:00–23:59	1705	6.93	1506	9.09	199	2.48
Year	2014	3735	15.18	2291	13.82	1444	17.98
	2015	4307	17.50	2741	16.54	1566	19.50
	2016	8091	32.88	5714	34.48	2377	29.60
	2017	8472	34.43	5828	35.16	2644	32.92

**Table 2:** Incidence–density ratio (IDR) estimates for the association between helmet use and death of cyclists, based on Poisson regression models.

	Crude estimates		Adjusted estimates					
			For all variables^1^ except cycling area		Only for cycling area		For all variables	
	IDR	95% CI	IDR	95% CI	IDR	95% CI	IDR	95% CI
All sample	1.10	0.83–1.44	0.81	0.59–1.10	0.42	0.31–0.58	0.45	0.32–0.63
Urban areas	0.67	0.39–1.16	0.59	0.34–1.04				
Open roads	0.34	0.24–0.48	0.40	0.27–0.58				
*P* value^2^	0.024		0.223					

^1^Age, sex, nationality, commission of infractions, reason for cycling, type of crash, traffic lane characteristics, meteorological conditions, road surface, time of day, and year.

^2^*P* value of the interaction term between cycling area and helmet use.

## Discussion

In line with most previous studies^1,2^, our final results show an inverse relationship between cyclists’ helmet use and death. The magnitude of this association (IDR 0.45 after adjustment for all variables; i.e. risk reduction of 55%) was very similar from that observed in previous meta-analyses and not very different from that reported in Australia after helmet laws were introduced^11^. These estimates are important in the road safety area. To contextualize it, a recent meta-analysis has pointed out that seatbelts reduce fatal injuries by 44% among rear seat occupants^12^.

Although a causal interpretation cannot be ascribed to this association (as is the case for any observational study of this nature), it provides another piece of evidence in favour of the protective effect of helmets on the risk of a cyclist’s death after a crash. However, the main utility of our study is to stress the need for observational study designs to give careful consideration to the strong confounding or modifier effect of some variables which may be easily overlooked. Regarding the protective effect of helmet use, cycling area is an excellent example of a confounder. Although mandatory legislation such as that currently in effect in Spain, Israel, Chile or Slovakia^13^ might be a main determinant of differences in the prevalence of helmet use depending on cycling area, this is not the only cause. Several other studies have reported similar results, with the higher prevalence of helmet use on open roads^7,14–16^ explained by factors such as sport cycling and differences in risk perception. In addition, the association between the area where the crash occurred and its severity is well documented in previous studies^17–21^. However, few studies to date have specifically compared cyclists’ road safety on urban and open roads, including rural settings. Bambach et al.^6^ considered the effectiveness of helmet use against head injury in rural and urban locations in Australia, but their results were non-significant and the location was not included in the multivariate analysis. In Taiwan, Kuo et al.^22^ found that cyclists who sustained head injuries were cycling in the fast lane much more frequently than on rural roads (29.8% on rural roads vs. 2.8% on urban roads). This finding may be associated with crash severity, and thus with a fatal outcome. In Denmark, Kaplan et al.^23^ found severe injuries to be more frequent on rural roads than in dense urban settings. The authors hypothesized that safety in numbers might affect their associations, but it seems more plausible to attribute these differences to the speed of the vehicles involved in crashes. In Spain, speed limits in urban areas are set at 50 km/h maximum^24^. Rural areas include open roads which may allow speed limits of almost double (90 km/h). Lastly, although Aldred et al.^3^ explicitly recognized the possible confounding role of cycling area on their estimate of the association between helmet use and crash severity, it seems surprising that they did not control for this factor.

With respect to other confounders, our results also show that cyclist- and environment-related factors tend to mask the inverse relationship between helmet use and death (i.e., an IDR of 1.10 in the crude estimate vs. 0.81 after adjustment for all variables except cycling area). After adjustment for cycling area, these factors continue to bias the association away from the null, albeit to a very small extent (i.e., an IDR of 0.42 or 0.45). This pattern is consistent with that found in several previous studies^20,23,25–28^.

Regarding the hypothetical role of cycling area as an effect modifier, our results also provide evidence in support of this role, although the effect was smaller. The differences in our point estimates of IDR in the two strata defined by cycling area seem to point clearly to a stronger protective effect of helmet use on open roads, where collision speeds are likely higher. This pattern was evident in our crude IDR; however, the differences were smaller for the corresponding adjusted estimates given that their 95% CI overlapped, and the high *P* value does not allow us to rule out chance as the only explanation for these differences. Unfortunately, comparisons between these findings and previous studies are hampered by the lack of published studies on this topic. We identified only one similar study (from France), in which Amoros et al.^14^ found—as we did—that the protective effect of helmet use was much greater in rural areas than on urban roads. These authors identified an interaction between helmet use and area of the crash for the risk of severe injuries.

Apart from its observational design, other limitations related mainly with our data source may compromise the validity of our estimates. Our database is not supported by any coroner´s report, thus, the cause of death cannot be identified objectively, and it could have been due to other unrelated causes. Selection bias may arise because, as in any police-based register, less severe crashes are underrepresented^29,30^. If helmet use causes a true reduction in the severity of injuries, this would lead to underestimation of its protective effect in our study. Regarding information bias, although we used a multiple imputation procedure to reduce bias related to missing values for helmet use and some other variables, this strategy is not useful to control for biases when missing data are generated by a not-at-random mechanism (MNAR), a situation which is highly plausible for the undetermined number of missing values for helmet use.

In summary, our hypothesis regarding the possible confounding effect of cycling area on the association between helmet use and risk of death is supported by our results. The findings for the role of cycling area as an effect modifier, however, are less clear although our results point towards this effect. These results have two practical implications. First, we provide an excellent real-life example for teaching purposes in two topics that are highly relevant to epidemiology, e.g., confounding and effect modification, given that it is not easy to find a variable which can behave in both these ways. Second, our results stress the need to carefully address heterogeneity across observational studies in attempts to analyse the magnitude of effect of protective interventions such as helmet use. Our results draw attention to the need for road safety researchers to be alert to the potentially important effect that some easily overlooked variables may have on causal mechanisms inferred from estimates of the magnitude of association. Otherwise, the direction of the estimated associations could be incorrect, as in some of the studies mentioned above^3^. This would be an example of Simpson's paradox^31,32^.

## Methods

We analysed the case series comprising all 24,605 cyclists involved in road crashes in Spain from 2014 to 2017, as recorded in the Spanish Register of Victims of Road Crashes maintained by the Spanish National Directorate of Traffic^33^. Except for data from two regions (Catalonia and Basque Country), for which information is lacking for 2014 and 2015, this is a nationwide, anonymized police-based database that contains data on every crash recorded by the national police corps in which at least one person was injured. We excluded crashes that occurred in Ceuta and Melilla—Spanish cities located overseas that have no open roads. Because the database is anonymized and maintained by a third party, and there was no intervention, this study was exempt from the requirement to seek informed consent or ethics committee approval.

Our exposure variable was helmet use (yes/no), and our outcome was death within the first 30 days after the crash (yes/no). Other covariates were individual characteristics of the cyclists, and environmental variables. Further information and details on the selection of variables were reported previously^17^. These variables are summarized in Table 1.

The proportion of missing values for our exposure variable (helmet use) was greater than 25%. Assuming that a non-despicable proportion could have been missed due to a missing-at-random mechanism, we used the Stata´s command ICE^34^ to implement a multiple imputation procedure with 50 iterations according to the chained equations method proposed by Van Buuren^35^, as suggested by the existing literature^36^. We considered that there could be differences in our data nested in the province where the crashed occurred, so a multilevel model was used (with cyclist and province as aggregation levels). Because death was an infrequent outcome, we used Poisson regression modelling to obtain the incidence–density ratio with 95% confidence intervals (IDR; 95% CI) in order to quantify the magnitude of association between helmet use and the risk of death. The estimates for each imputed dataset were combined by applying Rubin´s method with the MIM command^34^. We obtained crude IDR estimates for the whole sample, and for two strata according to cycling area, and three additional IDR: adjusting (1) only for cycling area, (2) for the remaining variables which could act as confounders, and (3) for all variables. The *P* value for the interaction term between helmet use and cycling area was obtained in crude and adjusted models. All statistical analyses were done with Stata software v.14^37^.

## Data Availability

The data underlying this article were provided by Spanish National Directorate of Traffic by permission. Data will be shared on request to the corresponding author with permission of Spanish National Directorate of Traffic.
